# Response Guided Interferon Therapy for Genotype 3 of Chronic Hepatitis C: Compliance and Outcome

**DOI:** 10.12669/pjms.314.7293

**Published:** 2015

**Authors:** Shahid Sarwar, Anwaar A. Khan, Shandana Tarique

**Affiliations:** 1 Shahid Sarwar, FCPS (Medicine) FCPS (Gastroenterology) Associate Professor of Medicine Gujranwala Medical College Consultant Gastroenterologist Doctors Hospital and Medical Center (DHMC) Lahore; 2 Anwaar A. Khan, MACP, FACG, FRCP, AGAF, FCPS Ex- Dean and Professor of Gastroenterology, ShaikhZayed Post Graduate Medical Institute, Lahore, Pakistan Consultant GastroenterologistDHMC, Lahore, Pakistan; 3 Shandana Tarique, FCPS (Medicine) Associate Professor of Medicine, Gujranwala Medical College, Pakistan

**Keywords:** Chronic hepatitis C, Compliance, Genotype 3, Response guided therapy, Sustained viral response (SVR)

## Abstract

**Objective:**

To determine compliance and improvement in sustained viral response (SVR) by following response guided therapy (RGT) plan of interferon and ribavirin, for genotype 3 in chronic hepatitis C.

**Methods:**

Patients with chronic hepatitis C genotype 3, who were eligible for interferon-ribavirin therapy and consented for RGT, were included. Those with no rapid viral response (RVR), having coarse echotexture of liver or undergoing re-treatment, were advised 48 week treatment whereas, rest had 24 week standard therapy. PCR for HCV RNA checked 6 months after discontinuing treatment, was the primary end point of study.

**Results:**

Of 154 patients, included in the study with mean age of 39.9 (±10.84) and male to female ratio 1.4/1 (94/60), majority of patients, 136 (88.4%) were treatment naïve whereas, 18 (11.6%) were being retreated. On ultrasound, 63 (40.9%) patients had coarse liver and 33 (21.4%) had splenomegaly. RVR was achieved in 99 (64.3%) patients. Overall 66(42.8%) patients merited extended duration of therapy as per RGT plan but only 22 (33%) were compliant. Treatment related side effects were the dominant reason for declining RGT in 33 (75%) patients. SVR was noted in 111 (72.1%) patients. Those patients with extended therapy (RGT), had SVR 90.9% (20/22), although, better but statistically not significant than those who stopped therapy at 6 months 77.2% (34/44) (p value 0.11).

**Conclusion:**

Response guided therapy plan did not improve SVR to pegylatedinterferon and ribavirin therapy in patients with genotype 3 and it has low patient compliance due to treatment related side effects.

## INTRODUCTION

Hepatitis C continues to be a challenge for human race due to its increasing prevalence and its potentially lethal long term complications.^1^ Ever since identification of this virus, efforts to find effective treatment are underway and a lot has been achieved i.e., combination therapy, pegylated interferon with ribavirin to directly acting antiviral (DAA) agents, introduced only recently.^2^

Due to prohibitive cost of DAA agents, interferon is likely to continue as predominant form of treatment available to patients with hepatitis C particularly, in the developing countries like Pakistan.^3^,^4^ Despite having genotype 3 as dominant variety in our population, viral clearance as determined by sustained viral response (SVR) is around 65-70%, less than what is reported from other regions of the world with this genotype.^5^ Moreover, standard interferon therapy which is no more standard of care as per international guidelines and achieves even lesser viral clearance as compared to pegylatedinterferon, is still the dominant form of treatment available to our patients.^6^

In order to enhance results of combination therapy, response guided therapy (RGT) is recommended by all major societies of liver diseases.^7^ RGT is aimed at improving viral clearance by prolonging treatment in patients with slower response to interferon therapy during initial part of therapy. Number of studies have shown promising results with response guided treatment plan with improvement in SVR,^8^,^9^ although, it adds to cost of treatment in form of additional investigations and more medications.

This study addresses the impact of RGT in our population as we still are unaware of true merits of this regime in our patients with chronic hepatitis C. Moreover, it highlights the issue of compliance of injection therapy in our patients due to side effects associated with use of interferon.^10^ Objectives of our study, therefore, were to determine patient acceptance and impact on outcome in terms of sustained viral response of RGT, in genotype 3 patients in our population.

## METHODS

Patients included in the study were those coming to Hepatology Clinic at the DHMC, Lahore from November 2011 to March 2014. Only confirmed cases of chronic hepatitis C as determined by positive polymerase chain reaction (PCR) test for HCV RNA, with lower limit of detection as 10 IU/ml and genotype 3, were included. Patients with evidence of decompensated liver disease i.e. presence of ascites on ultrasound examination, history of hepatic encephalopathy or gastrointestinal bleeding due to esophagealvarices were excluded. Patients with platelet count less than 80,000/mm^3^, International normalization ratio (INR)> 1.5, serum albumin < 3.2 gm/dl, serum creatinine> 1.5 mg/dl or deranged thyroid profile were not included. Presence of co-morbid illnesses i.e. uncontrolled hypertension, diabetes mellitus, unstable cardiac failure, stroke, major depression were excluded

Choice of interferon to be used (standard vs pegylated) was made after consultation with patients in view of their economic status. Patients were explained response guided therapy plan as suggested in guidelines of Asia Pacific Association for Study of Liver Diseases (APASL).^11^ Informed consent was obtained on start of study.

Patients were treated with pegylated interferon alpha 2a, 180µg subcutaneously on weekly basis with oral ribavirin 15 mg/kg/day. Those opting for standard interferon, received 3 million units thrice a week along with above dose of ribavirin. All patients were followed-up initially at fortnight, and then monthly till completion of treatment, thereafter, 3 monthly for 6 months to determine SVR. Complete blood count and liver function tests were checked on each follow up visit, along with evaluation and management of treatment related side effects. Patients had their quantitative HCV RNA status checked by real time PCR after one month of treatment to determine rapid viral response (RVR). If RVR was achieved, patients were advised to complete 6 month treatment. In case of positive PCR at one month, real time quantitative PCR for HCV RNA was repeated after 12 injections. Patients with negative PCR at 12 weeks (Complete Early viral response cEVR) or positive PCR but more than two log decline of viral load as compared with baseline viral load (Partial EVR), were advised to continue treatment for one year while those with less than 2 log decline in viral load after 3 months therapy were advised to discontinue therapy.

Patients not willing to continue therapy after six months despite indication were again counseled regarding need for extended therapy. Once treatment completed, HCV RNA was determined by PCR to document end of treatment response (ETR). Patients with positive PCR were non-responders whereas, those with negative PCR at end of treatment were checked again after 6 month to document sustained viral response (SVR) or relapse.

### Statistical analysis

Data were analyzed using SPSS 20. Numerical variables were described as mean ± standard deviation or median value. Categorical variables were given as percentage. Patients who opted for extended treatment and those declining treatment extension, were compared using unpaired two tailed student’s t test for numerical variables and chi square χ^2^ for nominal and categorical variables.

## RESULTS

Of the 154 patients included in the study, mean age was 39.9 (±10.84) whereas, male to female ratio was 1.4/1 (94/60). Genotype was 3a in 152 (98.7%) and 3b in two patients. Majority of patients, 136 (88.4%) were treatment naïve, whereas, 18 (11.6%) were non-responders to standard interferon therapy in past. On ultrasound examination 63 (40.9%) patients had coarse texture liver and 33 (21.4%) had splenomegaly.

Pegylated interferon along with ribavirin was started in 106 (68.8%) patients whereas, 48 (31.2%) opted for standard interferon and ribavirin. All patients had their HCV RNA checked by PCR at one month interval, after starting treatment, to determine rapid viral response (RVR). HCV RNA was undetectable at one month in 99 (64.3%) of patients whereas, 55 (35.7%) failed to achieve RVR. Among 55 patients with no RVR, only 18 (32.7%) had extension of treatment duration. Only 2 (11.1%) of 18 treatment experienced patients, had continued treatment beyond 6 months. Of 63 patients with coarse liver on ultrasound, 50 (72.4%) were eligible for extended therapy but only 13 (26%) were treated beyond 6 months with combination therapy. Overall 66 (42.8%) patients merited extended duration of therapy as per RGT plan but only 22 (33%) received RGT. Of 44 patients whose therapy was not extended despite indications, 33 (75%) declined RGT due to side effects of treatment and 11 (25%) due to non-affordability. Treatment failure was declared at 6 months in 25(16.2%) patients, of which, 11 were non-responders, 9 stopped treatment at three months as failed to achieve even partial early viral response (pEVR) and in 5, treatment was discontinued earlier than 6 months due to treatment related side effects. Duration of treatment was six months in 118 (76.6%) patients whereas, 14 (9.1%) stopped before 6 months. Of 22 patients with extended therapy, 18 (81.8%) received 12 month treatment, 3 (13.6%) could continue treatment up to 9 months and 1 stopped at 10 months of therapy due to intolerance from side effects.

End of treatment response (ETR) was achieved in 125 (81.7%) patients whereas, 24 (15.6%) were non-responders and 5 failed to complete treatment. Sustained viral response was noted in 111 (72.1%) patients and 14 (9%) patients had relapse. SVR was better in treatment naïve patients as compared with those receiving retreatment, 75.7% (103/136) vs 44.4% (8/18) (p value < 0.005). SVR was also better in those who achieved RVR as compared with those with positive PCR at 1 month, 81.8% vs 54.5% (p value < 0.001).Response rate was not different among patients on standard interferon than those on pegylated interferon therapy 66.6% vs 72.5% (p value 0.31). We compared patients with and without SVR as shown in Table-I.

**Table-I:** Comparison of patient with SVR and those with no SVR.

Variables	Patients with SVR (n- 111)	No SVR (n- 43)	P value
Mean Age(years)	38.59 (10.6)	43.47 (10.7)	0.012
Mean Baseline ALT (U/L)	93.76 (78.3)	100.4 (60.4)	0.61
Mean Serum albumin g/dl	4.1 (0.39)	4.04 (0.47)	0.32
Platelet (x 10^9^/L)	190.4 (64.4)	199.3(93.2)	0.5
No of Patients with Coarse liver texture	41	22	0.25
No of patients with Splenomegaly	21	12	0.23
No of Patients with RVR	81	18	< 0.001

Biochemical response with normal alanine aminotransferase (ALT), at completion of therapy was noted in 125 (81.5%) patients, 22 (14.3%) showed decline in ALT, but it was not within normal limits whereas, 7 (4.5%) had no change or raised ALT at end of treatment. Major side effects experienced were body ache and myalgia in 76 (49.4%), drop in hemoglobin below 10 g/dl in 51 (33.1%), decline in platelet count in 106 (68.8%) patients and depressive symptoms in 10 (6.5%) patients. Erythropoietin was needed in 40 (26%) patients for anemia during treatment.

Impact of treatment extension on SVR, due to different indications is shown in Table-II. Of those patients, who met indications for extended treatment, SVR with response guided therapy was 90.9% (20/22), better, but statistically not significantly superior, than SVR in patients who stopped therapy at 6 months despite indication 77.2% (34/44) (p value 0.11).

**Table-II:** SVR in patients with indication for extended therapy as per Response guided therapy.

Indication for extension	Treatment extended SVR (%)	Treatment not extended SVR (%)	P value
Coarse texture liver	92.3	78.3	0.26
No RVR	89.4	81.5	0.78
RVR but coarse liver	100	74.07	0.11
All patients	90.2	77.2	0.11

## DISCUSSION

Genotype 3 is the most underrated and poorly understood type of hepatitis C virus. For years, we regarded it as good genotype with favorable outcome of interferon therapy. But majority of studies from Asian region have shown lower SVR as compared with western data. Batool et al.^12^ noted 68% response rate in genotype 3 whereas, it was around 72% in a study of 721 patients from Lahore.^6^ Niederau C et al. concluded that patients with genotype 3 have more fibrosis of liver and only 56.9% of them respond to interferon therapy.^13^ SVR of 72.1% in our study patients corroborates with the other reported studies.^14^ It is disturbing to see that new emerging directly acting drugs (DAAs) with promise of interferon free treatment of hepatitis C are reported to be less effective in genotype 3.^15^ Impact of genotype 3 on insulin resistance and lipid metabolism is presumed to be one factor responsible for its poor response to treatment.^16^

In order to address this issue of inadequate response, response guided therapy was introduced. Rapidity of viral disappearance RVR, is directly related to viral clearance,^8^ also seen in this study. SVR in patients with RGT was above 90%, better than response in patients who refused extended therapy despite indications (77%), but the difference was not statistically significant. It is most likely due to small number of patients on RGT plan. Fried MW et al noted that patients with RVR have better SVR ranging from 88-100% across all genotypes,^17^ but SVR in patients without RVR is low, 45% reported by Shiffman ML,^18^ and 56% in Dalgard O study,^19^ with 24 week treatment. It is due to less than adequate response in patients failing to achieve RVR, that both EASL^7^ and APASL^11^ guidelines recommend, extension of treatment duration to 48 weeks in genotype 3. Although, our study fails to show this benefit of RGT, SVR in patients with RGT was better, (> 90%) than in patients with RVR treated for 24 weeks (81.8%). If number of patients on RGT is higher, it may become statistically significant, as the trend shows. Ruddy KR stresses the importance of identifying indicators of poor response in addition to no RVR i.e. African origin, cirrhosis, unfavorable IL28B and decreased interferon sensitivity to maximize benefits of RGT.^20^

Our study brings forth the issue of compliance and acceptability of RGT, among patients. Only 33% of patients, needing extended therapy, continued treatment beyond 6 months. Despite, agreeing with RGT plan at start of treatment, majority of patients refused treatment extension due to treatment related side effects. In as much as, adverse events tend to increase over time, 48 week treatment becomes difficult to tolerate as compared with 24 week therapy.^21^ Side effects i.e. fatigue, weight loss, myalgia and depression do affect quality of life adversely, thus, limiting therapy from extending to 48 weeks.^22^

Response guided interferon therapy can marginally improve the response rate to interferon therapy as seen in our study, but due to poor compliance of extended therapy, number of patient on RGT were limited, it merits a larger case control study to see better results. Due to prohibitively high price of DAAs, these will not be affordable for majority of our population; it is therefore, prudent to further explore RGT and such other measure to treat patients with Hepatitis C in Pakistan.

## CONCLUSION

Response guided therapy plan does not improve response to peg interferon and ribavirin combination therapy in patients with HCV genotype 3 and it has suboptimal patient compliance due to treatment related side effects.
